# Dynamic response in the larval geoduck (*Panopea generosa*) proteome to elevated *p*CO_2_

**DOI:** 10.1002/ece3.5885

**Published:** 2019-12-06

**Authors:** Emma Timmins‐Schiffman, José M. Guzmán, Rhonda Elliott Thompson, Brent Vadopalas, Benoit Eudeline, Steven B. Roberts

**Affiliations:** ^1^ Genome Sciences University of Washington Seattle WA USA; ^2^ School of Aquatic and Fishery Sciences University of Washington Seattle WA USA; ^3^ Taylor Shellfish Hatchery Quilcene WA USA; ^4^ Mason County Public Health Shelton WA USA; ^5^ Washington Sea Grant University of Washington Seattle WA USA

**Keywords:** data‐dependent acquisition, geoduck, mass spectrometry, mollusk, ocean acidification, pCO_2_, proteome, shellfish

## Abstract

Pacific geoducks (*Panopea generosa*) are clams found along the northeast Pacific coast where they are important components of coastal and estuarine ecosystems and a major aquaculture product. The Pacific coastline, however, is also experiencing rapidly changing ocean habitat, including significant reductions in pH. To better understand the physiological impact of ocean acidification on geoduck clams, we characterized for the first time the proteomic profile of this bivalve during larval development and compared it to that of larvae exposed to low pH conditions. Geoduck larvae were reared at pH 7.5 (ambient) or pH 7.1 in a commercial shellfish hatchery from day 6 to day 19 postfertilization and sampled at six time points for an in‐depth proteomics analysis using high‐resolution data‐dependent analysis. Larvae reared at low pH were smaller than those reared at ambient pH, especially in the prodissoconch II phase of development, and displayed a delay in their competency for settlement. Proteomic profiles revealed that metabolic, cell cycle, and protein turnover pathways differed between the two pH and suggested that differing phenotypic outcomes between pH 7.5 and 7.1 are likely due to environmental disruptions to the timing of physiological events. In summary, ocean acidification results in elevated energetic demand on geoduck larvae, resulting in delayed development and disruptions to normal molecular developmental pathways, such as carbohydrate metabolism, cell growth, and protein synthesis.

## Graphical abstract

The physiology of geoduck clam larval development at two different pH was tracked over days 6–19 postfertilization using global proteomic profiling. Proteomic profiles revealed that some molecular profiles were maintained, despite differences in pH, while others were altered by a change in environment (e.g., metabolism, cell cycle, protein turnover).

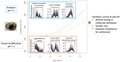

## 1 INTRODUCTION

The Pacific geoduck (*Panopea generosa*) is a burrowing hiatellid clam found in low intertidal and subtidal sediments throughout the northeast Pacific coast, including the United States (Alaska, Washington, and California), Canada (British Columbia), and Mexico (north Baja Pacific Coast) (Coan, Scott, & Bernard, 2000; González‐Peláez, Leyva‐Valencia, Pérez‐Valencia, & Lluch‐Cota, 2013; Vadopalas, Pietsch, & Friedman, 2010). This bivalve mollusk is one of the largest burrowing clams in the world, with specimens over 3 kg having been recorded (Goodwin & Pease, 1987). Geoducks are also among the longest‐lived organisms in the animal kingdom, with an average reproductive life span of 30 years (Sloan & Robinson, 1984) and living up to 163 years (Bureau et al., 2002). These clams are known to play key roles in maintaining ecosystem health via seston filtration and biodeposition, as well as being a food source for marine organisms, including sea otters, fishes, crabs, and sea stars (Newell, 2004; Straus, MacDonald, Crosson, & Vadopalas, 2013). In addition to its ecological importance, geoduck is the most economically important clam fishery in North America (Hoffmann, Bradbury, & Goodwin, 2000) and the target of a growing aquaculture industry in Puget Sound (Washington, USA) and British Columbia, with estimated annual production valued at US$30 million (SFW, 2016). Pacific geoducks are considered to be particularly vulnerable to ocean acidification due to their calcifying nature and biogeographical distribution. As marine calcifiers, geoduck relies on calcite and aragonite for shell secretion (Orr et al., 2005; Weiss, Tuross, Addadi, & Weiner, 2002), both of which become less biologically available as pH declines (Feely, Sabine, Hernandez‐Ayon, Ianson, & Hales, 2008). Additionally, Puget Sound and marine waters just off the Washington coast are naturally at lower pH than those of other regions (Busch, Harvey, & McElhany, 2013), and ocean acidification has already caused a local decrease in pH of 0.05–0.15 units since the Industrial Revolution (Feely et al., 2010). Despite the ecological and economic importance of this giant clam in North America, the potential effect of ocean acidification on the physiology of the Pacific geoduck at its different life stages remains elusive.

The physiological impacts of ocean acidification on marine shelled mollusks during early life stages have been addressed in a number of species (e.g., Frieder, Applebaum, Pan, Hedgecock, & Manahan, 2016; Kapsenberg et al., 2018; Ko et al., 2014; Stumpp et al., 2013; Timmins‐Schiffman, O'Donnell, Friedman, & Roberts, 2013; Waldbusser et al., 2013). It is generally observed that low pH affects somatic growth and shell production in larval and juvenile shellfish and alters respiration, feeding and excretion rates, energy availability and overall fitness. In exposures to decreased pCO_2_, bivalve larvae are generally negatively affected, with impacts on hatching rate, growth, metamorphosis, and decreased calcification rates (Huo et al., 2019; Miller, Reynolds, Sobrino, & Riedel, 2009). Juvenile bivalves have similarly demonstrated decreases in calcification rates, shell dissolution and malformation, and impacts on feeding, respiration, and energy storage when exposed to low pCO_2_ (Beniash, Ivanina, Lieb, Kurochkin, & Sokolova, 2010; Dickinson et al., 2012; Dove & Sammut, 2007a, 2007b; Fernández‐Reiriz, Range, Álvarez‐Salgado, & Labarta, 2011; Gazeau et al., 2007; Michaelidis, Ouzounis, Paleras, & Pörtner, 2005). While these and other studies provide critical information on how changes in seawater chemistry driven by ocean acidification are altering the physiological performance and ecology of marine shelled mollusks, the molecular mechanisms, and pathways underlying the impacts of ocean acidification throughout the time course of molluscan larval development are unknown.

The proteome is closer to the realized phenotype than the genome or the transcriptome as proteins are the molecules that interact with the environment and are under direct selective pressure. Mass spectrometry (MS)‐based proteomics technology provides a large‐scale, unbiased approach for examining relative abundances of all proteins present in a given sample (e.g., Timmins‐Schiffman et al., 2017). In a previous study, the response of the Pacific oyster larval proteome to ocean acidification was surveyed using low‐resolution, 2‐dimensional electrophoresis coupled with mass spectrophotometry (Dineshram et al., 2012). The authors found that 18.7% of the proteins detected in control samples (379 proteins total) were inhibited after exposure to a lower pH, including a severe inhibition of proteins related to calcification and cytoskeleton production. In proteomics studies of *Crassostrea hongkongensis* and *C. gigas* pediveligers exposed to ocean acidification, proteomic shifts in response to low pH were detected in proteins involved in the antioxidant response, translation, structural molecules, and metabolism (Dineshram et al., 2016, 2015). Using a different proteomic approach, Spencer et al. (2019) evaluated the effect of natural pH variation on the proteome in juvenile geoduck. In a targeted proteomics assay of 13 environmentally sensitive proteins, no difference in abundance was found between habitats/pH regimes, suggesting that juvenile geoduck may tolerate a wide range of pH. However, the effect of ocean acidification during earlier, more sensitive developmental stages or at other physiological processes (e.g., metabolism, biomineralization, cytoskeleton) remains unknown.

In this study, we use a global, high‐resolution mass spectrometry‐based proteomics data‐dependent analysis to (a) characterize for the first time the proteome of geoduck during early life stages (6 through 17 days postfertilization) and (b) evaluate the physiological impact of a lower environmental pH at the proteome level. We hypothesize that larval stages of geoduck will be susceptible to the impacts of ocean acidification, similar to other pelagic bivalve species. We expect to see a significant proteomic shift in response to pH, especially in proteins involved in the oxidative stress response, cytoskeleton restructuring, metabolic pathways, and calcification.

## 2 METHODS

### 2.1 Larval rearing

**Figure 1:**
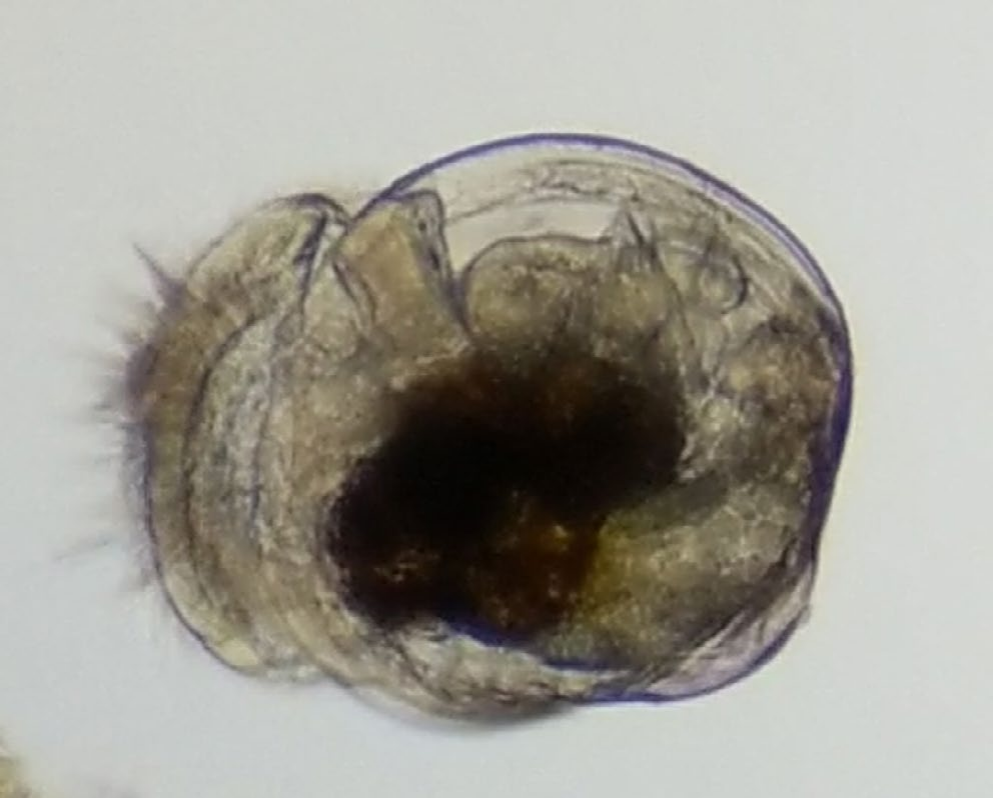
A Pacific geoduck larva

Pacific geoduck (*Panopea generosa*) broodstock maturation, spawning, and early larval development (days 1–4 postfertilization) occurred in seawater buffered with Na_2_CO_3_ (pH 8.2) in a commercial hatchery in Hood Canal, WA per normal operation conditions. At day 5 postfertilization, larvae (Figure 1) were exposed to ambient pH (7.5) or decreased pH conditions (7.1) in 200‐l conical tanks (two tanks for each condition). The pH of 7.5 is the ambient pH of untreated incoming water to the hatchery, whereas the pH of 7.1 was obtained by adding CO_2_ with a solenoid valve and venturi injector with a 5,200 YSI probe for monitoring. Water was maintained at approximately 14°C throughout the experiment which ended 19 days postfertilization. Initial stocking density of each conical tank was 70,000 fertilized eggs/L.

Larvae were sampled on days 6, 8, 10, 12, 14, and 17 postfertilization for proteomic analysis. Specifically, approximately 10,000 larvae from each tank (two at pH 7.1 and two at pH 7.5) were collected using a 20‐µm mesh screen, rinsed with 70% isopropyl alcohol, and stored at −80°C. For each sampling event, larval size classes were determined using a series of mesh screens (from 90–200 µm in 20‐µm intervals). The shell lengths of larvae (*n* = 50) at each sampling time point were measured to determine average larval size per day and treatment. Differences in average larval size between the factors treatment, age, and their interaction were assessed using a nested ANOVA in the R (R Core Team, 2016) package lme (Pinheiro, Bates, DebRoy, Sarkar, & Core Team, 2016) with larval tank of origin as a random effect. The number of larvae retained in each screen was estimated by weight (to 0.0 g) based on an established conversion developed in the hatchery (Appendix S3). Larval counts were averaged across conical tanks. Larval settlement competency was determined when foot extension and movement were present in at least 30% of larvae on the screen.

The effect of pH on larval size was assessed used a chi‐squared test for homogeneity with a *p*‐value cutoff of .05 for days 10 and 17 postfertilization. Larvae were visually inspected for abnormalities on day 19.

### 2.2 Protein extraction and LC‐MS/MS

Larval proteins were digested, and peptides were desalted following Timmins‐Schiffman et al. (2014). Larval peptides were analyzed on an Orbitrap Fusion Lumos mass spectrometer (Thermo Scientific) with a 4.5 cm, 100‐µm precolumn and a 26 cm, 75‐µm analytical column, both packed in‐house with 3 µm C18 Dr. Maisch (Germany) beads. The analytical column was housed in a 50°C column heater for the duration of the analysis to improve chromatography. Over a 120 min method, a 90‐min acetonitrile gradient went from 5%–30%. In MS1 analysis in the Orbitrap, the resolution was 120 K, scan range was 375–1,575 *m/z*, max injection time was 50 ms, and AGC target was 700,000. During the MS2 analysis in the IonTrap maximum injection time was 100 ms, AGC target was 2,000, and centroided data was collected. The mass spectrometry proteomics data have been deposited to the ProteomeXchange Consortium via the PRIDE (Perez‐Riverol et al., 2019) partner repository with the dataset identifier PXD013667.

### 2.3 Proteomics analysis

All mass spectrometry raw files were searched against a deduced geoduck larvae proteome with the addition of common laboratory contaminants from bovine, human, and other (https://crapome.org). Redundancy of sequences in the larval proteome was reduced using CD‐hit (Li, Jaroszewski, & Godzik, 2001). The protein database was translated from the sequenced transcriptome (NCBI BioProject Accession # PRJNA529226). Details on reference proteome development are described elsewhere (https://github.com/sr320/paper-Pg-larvae-2019). All files were searched against the database using Comet 2016.01 rev. 3 (Eng, Jahan, & Hoopmann, 2012; Eng et al., 2015) with parameters including concatenated decoy search, mass tolerance of 20 ppm, 2 allowed missed trypsin cleavages, fragment bin tolerance of 1.0005 and offset of 0.4. Peptide and Protein Prophet (Keller, Nesvizhskii, Kolker, & Aebersold, 2002; Nesvizhskii, Keller, Kolker, & Aebersold, 2003) were run consecutively using xinteract with no probability cutoff to allow for FDR cutoff later in the pipeline. Resulting pep.xml files were analyzed in Abacus (Fermin, Basrur, Yocum, & Nesvizhskii, 2011) with a FDR cutoff of 0.01 (probability from combined prot.xml file of 0.9).

Nonmetric multidimensional scaling (NMDS) analysis was performed on all proteins that were inferred across technical mass spectrometry replicates with at least two unique peptide spectral matches using the vegan package (Oksanen et al., 2016) in R. Protein abundance data (normalized spectral abundance factor) were log (*x* + 1) transformed, and a Bray–Curtis dissimilarity matrix was calculated. Eigenvectors were generated using envfit in the biostats package (McGarigal, 2009). An eigenvector was considered significant if its axis loading value was >0.9 and its *p*‐value was <.001. Proteins corresponding to significant loadings on each axis in each NMDS were subjected to enrichment analysis following Timmins‐Schiffman et al. (2017) at the following project‐specific portal: https://meta.yeastrc.org/compgo_emma_geoduck_larvae/pages/goAnalysisForm.jsp. ANOSIM was performed on normalized spectral abundance factor (NSAF) data standardized by row to assess significance of trends observed on the NMDS based on day and pH.

Differentially abundant proteins between consecutive time points (e.g., day 6 vs. day 8 within pH condition) were determined using QSpec (Choi, Fermin, & Nesvizhskii, 2008). Due to potential differences in developmental rate, differential protein abundances between treatments were not analyzed. For each QSpec analysis, only proteins that had a nonzero sum of spectral counts across replicates were included and spectral counts were summed across technical replicates.

In order to identify protein groups that had roles at specific time points during development, proteins with similar abundance trends were grouped using a hierarchical clustering method within each pH treatment. NSAF values were averaged across conical replicates within days, and a Bray–Curtis dissimilarity matrix was calculated in R to use with the average clustering method. Cluster division was based on dendrogram topology (cutoff of *h* = 0.5). Clusters were further classified into five categories based on the general trends through time determined with loess smoothing of each cluster. Clusters including at least 10 proteins were subjected to enrichment analysis following Timmins‐Schiffman et al. (2017). All R code used for analyses is provided at https://github.com/sr320/paper-Pg-larvae-2019.

## 3 RESULTS

### 3.1 Larval phenotype

**Figure 2:**
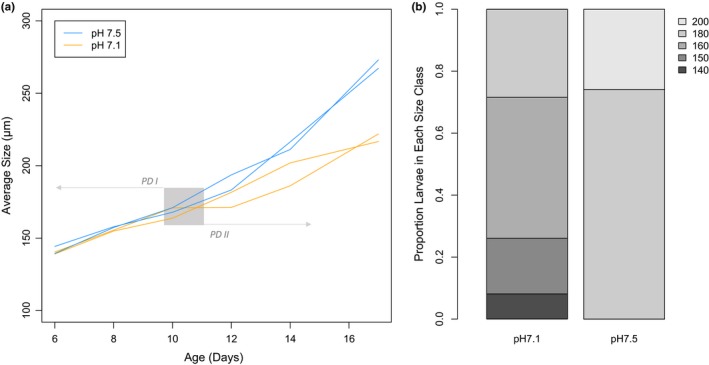
Growth curves of larvae from day 6 through 17 of development (a) and proportion of larvae in each size class (µm) on day 17 at pH 7.1 and 7.5 (b). Each individual tank in which larvae were reared is represented by a single line in panel a. Larval growth curves in tanks at pH 7.1 are orange and pH 7.5 tanks are in blue. The gray area indicates the larval transition from prodissoconch I (PD I) to II (PD II) (Goodwin & Pease, 1989)

At day 10 of the experiment, there was no observed difference in larval abundance between treatments, both having 2.3 million larvae. There was a difference in proportion of larvae in different size classes at day 10 (Χ^2^ = 50,021, degrees of freedom = 3, *p* < 2.2e‐16), but overall larval size was comparable at this time point between treatments (Figure 2; Appendix S1). By day 17, there was a clear difference in phenotype with larvae at pH 7.5 attaining a larger size (size classes 180 µm and 200 µm) than larvae at pH 7.1, which did not exceed 180 µm (Χ^2^ = 264,350, degrees of freedom = 4, *p* < 2.2e‐16) (Figure 2). Larval size was impacted significantly by larval age (ANOVA *p*‐value <.0001) and treatment × age (*p* = .032), but not by treatment alone (*p* > .05). However, the larvae at pH 7.5 experienced a significant mortality by day 17. Specifically, a 29‐fold increased mortality was observed at pH 7.5 compared to larvae at pH 7.1 (data not shown). Ciliates were detected at pH 7.5 at 10‐ and 14‐days postfertilization, but not at pH 7.1. At day 19, settlement was observed for larvae at pH 7.5 but not for larvae at pH 7.1.

**Figure 3:**
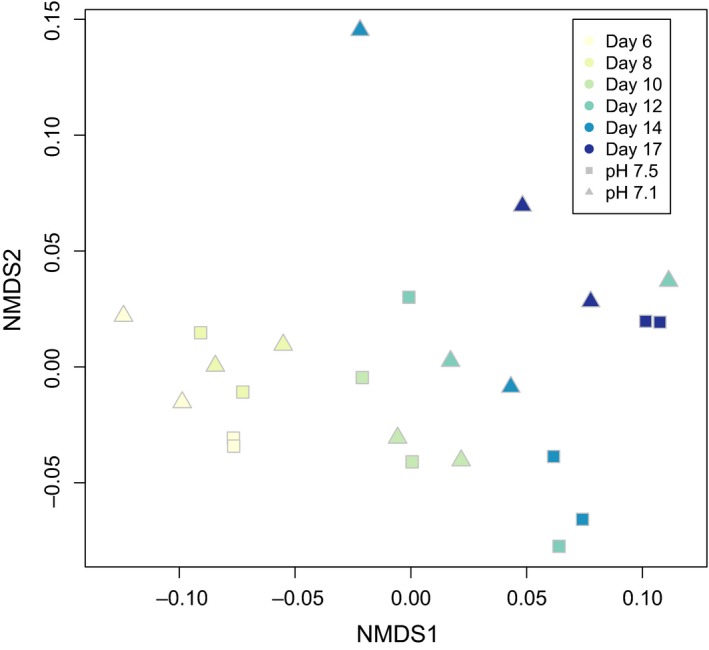
Nonmetric multidimensional scaling (NMDS) of larval proteomes across time and pH treatments. Increasingly dark colors correspond to progression through time (day 6–17). Squares represent proteomes from larvae reared at pH 7.5 and triangles represent larvae at pH 7.1

Across days 6 through 17 postfertilization and at both pH treatments, 6,328 proteins with at least 2 unique peptides across all biological and technical replicates were characterized (Appendix S4). In examining global protein abundance, there was a clear influence of time with significant separation of larval proteomes by day (ANOSIM *R* = 0.6579, *p* = .001), but not by pH (*R* = 0.008207, *p* = .338) (Figure 3). Proteomes are separated by time mostly along axis 1 of the NMDS, therefore, proteins with significant, positive eigenvector loadings along this axis (Appendix S2) play a dominant role in proteome differentiation over developmental time. The proteins with positive loadings are enriched for Gene Ontology (GO) Biological Processes carbohydrate metabolic process, cellular amino acid metabolic process, and monocarboxylic acid metabolic process (https://github.com/sr320/paper-Pg-larvae-2019).

**Figure 4:**
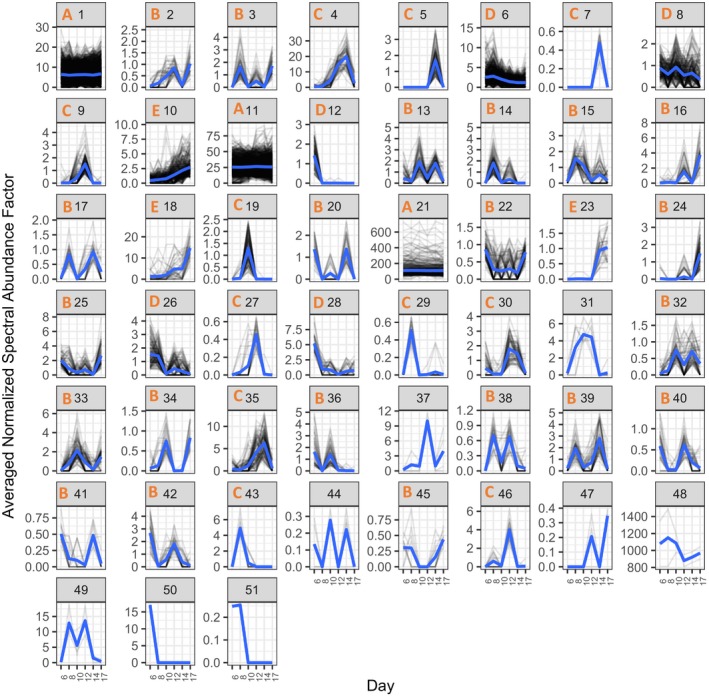
Plots of protein abundances of all proteins detected in pH 7.5‐reared larvae from days 6–17. Proteins are divided among plots based on hierarchical clustering of abundance patterns across time, with normalized spectral abundance factor on the *y*‐axis and time (days) on the *x*‐axis. Loess smoothed curves are represented by blue lines and abundance topology type is indicated by the orange letter at the top of each plot window for clusters with at least 10 protein members. Cluster number is indicated at the top of each plot window in black

**Figure 5:**
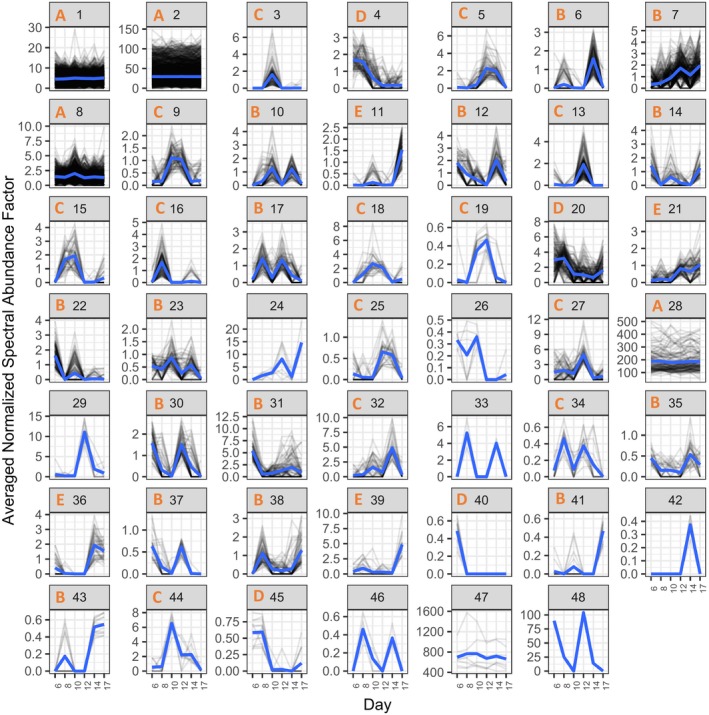
Plots of protein abundances of all proteins detected in pH 7.1‐reared larvae from days 6–17. Proteins are divided among plots based on hierarchical clustering of abundance patterns across time, with normalized spectral abundance factor on the *y*‐axis and time (days) on the *x*‐axis. Loess smoothed curves are represented by blue lines and abundance topology type is indicated by the orange letter at the top of each plot window for clusters with at least 10 protein members. Cluster number is indicated at the top of each plot window in black

The proteomic data from larvae reared at pH 7.5 clustered into 51 groups (43 of which had at least 10 protein members) and larvae reared at pH 7.1 clustered into 48 groups (40 of which had at least 10 protein members) based on abundance patterns over time (day 6 through 17) (Figures 4 and 5; Appendix S4). Cluster topology determined with loess smoothing resulted in five types of abundance patterns over days 6–17 postfertilization: stable/flat line (A), multiple peaks in abundance (B), single peak in abundance (C), general decrease over time (D), or general increase over time (E) (Table 1).

**Table 1:** Number of proteins (and percentage of total proteins at the given pH) that fall into each cluster type (A–E) across clusters at each pH. Proteins that are in clusters of fewer than 10 protein members are not categorized in a cluster type and so are not included

Cluster type	Number of proteins pH 7.5	Number of proteins pH 7.1
A: Stable/flatline	3,678 (51%)	4,601 (63%)
B: Multiple peaks in abundance	1,242 (17%)	949 (13%)
C: Single peak in abundance	651 (9%)	759 (10%)
D: General decrease over time	1,339 (18%)	292 (4%)
E: General increase over time	391 (5%)	268 (4%)

In an effort to define biological processes influenced by pH, proteins were grouped into three general categories, including proteins that show stable abundance across time and are not altered by pH; proteins that change in abundance during development but show similar trends at pH 7.5 and 7.1; and proteins that change in abundance during development in patterns specific to a pH treatment.

### 3.2 Basic cellular housekeeping is maintained across pH

Many of the protein functional groups that correspond to molecular housekeeping functions and that showed stable abundances across time (abundance pattern A) were maintained across both pH. The protein clusters that contain “housekeeping” proteins had high percentages (>50%) of shared protein members across pH treatments. These protein categories can be loosely described as ribosomes, translation, actin and myosin, proton‐transporting ATP synthase, protein folding, and tricarboxylic acid cycle (e.g., pH 7.5, cluster 21 and pH 7.1 clusters 1 and 2; Appendices S5 and S6). Many of these proteins had high abundances and likely represent the core housekeeping proteins that allow other physiological processes to function.

### 3.3 Some stage specific proteins are robust to pH

Many protein groups displayed time‐specific patterns of abundance, suggesting discrete roles at specific developmental time points. Our analysis allowed for comparison of broad protein functional categories across abundance patterns to discern trends of physiological activity at different time points. Many of these development‐specific patterns were shared between pH 7.5 and 7.1 on the broad scale of functional category, suggesting essential molecular checkpoints that are resistant to alteration during a low pH response, at least given the timing of our sampling scheme. Since we did not have daily sampling for proteomics, we cannot verify the exact timing of when certain processes “turned on” or “off,” making it impossible to definitively determine the coordination (or discoordination) of the timing of molecular events between pH. Additionally, an impact of pH on physiology may be reflected in the differences in individual proteins associated with the similar functional GO categories between the two environments.

At day 10 postfertilization, there was a signal of an upregulation of cytoskeletal proteins at both pH 7.5 and 7.1, different from those that maintained stable abundance (see above). At pH 7.5, the DAPs at higher abundance on day 10 were driven by the changing abundances of several tubulins (Appendix S4). In pH 7.1‐reared larvae, cytoskeletal proteins were at higher abundance on day 10 compared to both days 8 and 12, with the enrichment of the term structural constituent of cytoskeleton (molecular function—MF) in the comparison with day 8 and dynein complex (cellular component—CC) in the comparison with day 12 (Appendix S7).

Based on the proteomic data at both pH, some signals of the transition between prodissoconch I and II around day 12 are proteins related to cell division, transcription, and translation. Proteins supporting DNA replication were at increased abundance at day 12. At pH 7.5, this included the cluster 46 enriched term chromatin (CC) (Appendices S4 and S5). Similarly, DAPs were involved in DNA replication and were at increased abundance at pH 7.5 on day 12 compared to 14 (Appendix S4). At pH 7.1, the DAPs were enriched for methyltransferase activity (MF) (in a comparison with abundance at day 10) and terms related to telomere maintenance compared to day 14 (Appendix S7). Proteins involved in transcription and translation also increased in abundance at this time point, as reflected in the enriched terms associated with clusters 25 and 2 for pH 7.5 and 13, 21, 27, and 30 for pH 7.1 (Appendices S5 and S6).

Proteins involved in cellular growth and shell deposition were at increased abundance around day 14 postfertilization. Shell deposition‐related GO terms that were enriched at pH 7.5 included chitin metabolic process (biological process—BP) and chitin‐binding (MF) and at pH 7.1 were mucus layer (CC), chitin metabolic process (BP), and cell‐matrix adhesion (BP) (Appendices S5 and S6). Molecular signals of cell growth included the enrichment in the DAPs of the terms microtubule‐based movement (BP), dynein complex (CC), and motor activity (MF) at pH 7.5 and dynein complex (CC) at pH 7.1 (Appendices S7 and S8).

At day 17 postfertilization, DNA replication proteins again played an important role in the cluster and DAP analyses. Many DNA replication proteins were at increased abundance at pH 7.5, compared to day 14 (Appendix S4). At pH 7.1, the GO term centrosome (CC) was enriched in cluster 41 proteins (Appendix S6). The pH 7.1 DAPs (compared to day 14) were enriched for terms such as DNA conformation change (BP) (Appendix S7). In pH 7.1 cluster 14, proteins were enriched for the term regulation of transcription, DNA‐templated (BP), and the DAPs enriched for the term nucleic acid binding (MF) (Appendices S6 and S7).

### 3.4 Proteomic trends specific to pH 7.5

Proteomic trends specific to pH 7.5 included changes in abundance of cytoskeletal proteins during early larval stages (day 6 postfertilization), followed by increased levels of proteins involved in protein expression, cilia biogenesis, and transport (day 8). During the subsequent veliger stage, there was evidence of metabolic shifts in the proteomics data (days 10 and 12). In the late veliger stage, there was a surge in molecular markers associated with protein translation (days 14 and 17). Details are provided below.

At day 6, there was proteomic evidence of changes to larval cytoskeletal and membrane structures at pH 7.5. There was an increase in proteins associated with the GO term spindle pole (CC) (Appendix S5). Individual cytoskeletal proteins were at increased abundance compared to day 8, and the GO MF term phospholipid‐binding was enriched in the DAPs (Appendix S8).

On day 8, some of the predominant molecular functions that arose at pH 7.5 were post‐translational modifications, cilia biogenesis, and transport across cell membranes. In cluster 3, proteins were enriched for terms associated with post‐translational modification (Appendix S5). In cluster 39, the terms snRNA processing (BP) and integrator complex (CC) were enriched (Appendix S5). Cluster 14 was enriched for the GO term integral component of membrane (CC) (Appendix S5). Among the DAPs, the GO term motile cilium (CC) was enriched, based upon the increased abundance of three intraflagellar transport proteins (Appendix S8).

At day 10 postfertilization, there was a strong signal of a metabolic shift at pH 7.5. This shift was evidenced by an increase in abundance of proteins involved in glucose and fatty acid metabolism and protein degradation enzymes (Appendix S4). Proteins in clusters 19 and 32 had abundance peaks on day 10, with the former proteins enriched for the term phosphatidylinositol‐3‐phosphate binding (MF) and the latter enriched for hexokinase activity (MF) and glucose binding (MF) (Appendix S5).

On day 12, the term cellular glucan metabolic process (BP) was enriched in cluster 10 proteins, which increased in abundance over time (Appendix S5). More nuanced cellular processes, such as membrane budding, may have also been supported by this upregulation as seen in the proteins in cluster 9 that were part of the enriched term ESCRT I complex (CC).

On day 14 at pH 7.5, there was evidence of a large upregulation of transcription and ribosomal proteins. Clusters 4, 35, and 39 were enriched for GO terms related to translation (Appendix S5) due to the abundance of ribosomal proteins in these clusters. Among the DAPs that were at elevated levels on day 14, many were ribosomal and translation elongation factors leading to the enrichment of terms such as organonitrogen compound metabolic process (BP) and structural constituent of ribosome (MF) (Appendix S8). Proteins that decreased in abundance from day 14 to 17 were enriched for translation (BP) and ribosome biogenesis (BP) (Appendix S8).

On day 17, there was a decrease in abundance of ribosomal proteins (clusters 4 and 35 and DAPs), but an increase in abundance of proteins involved in post‐translational modification and in transport. Proteins in clusters 2, 3, and 24 had abundance peaks at day 17 and included enrichment for the GO terms Golgi membrane (CC), terms associated with post‐translational modifications, and integral component of membrane (CC) (Appendix S5). Similarly, the terms membrane budding (BP) and 1‐phosphatidylinositol binding (MF) were enriched in the DAPs on day 17 (Appendix S8).

### 3.5 Proteomic trends specific to pH 7.1

Proteomic changes specific to pH 7.1 included changes in abundance of transcription, DNA replication, and translation proteins (days 6 and 8). During mid‐veliger stage, there were increased abundances of protein transport proteins (day 10) and proteins involved in metabolic processes (day 12). On day 17, there was some evidence of a pH regulation response. Details for each day are provided below.

The molecular physiological profile of larvae reared at pH 7.1 on day 6 diverges from larvae reared at pH 7.5, in which the proteomic profile was dominated by cytoskeletal and membrane stability proteins. At pH 7.1, there was a strong signal of transcription‐ and translation‐related processes at this early time point, with many of the contributing proteins detected at high abundances at later points in development in pH 7.5 larvae. For example, the term large ribosomal subunit (CC) is enriched in cluster 4 proteins at pH 7.1 and includes three 60S ribosomal proteins, all of which are found in pH 7.5 clusters with marked peaks much later in development (clusters 35 and 4). In clusters 14 and 30, transcription‐associated processes were enriched, which included regulation of transcription, DNA‐templated (BP) and transcription factor activity, sequence‐specific DNA binding (MF) (Appendix S6). Other clusters (22, 37, and 40) with peaks at this time point were enriched for functions associated with cellular signaling, such as enzyme‐linked receptor protein signaling pathway (BP), neurotransmitter:sodium symporter activity (MF), and Notch signaling pathway (BP) (Appendix S6).

The proteins that increased in abundance on day 8 at pH 7.1 were mostly involved in DNA replication and translation. None of the proteins that came to the forefront for the pH 7.1 analysis had the same trend at pH 7.5. Cluster 16 proteins contributed to the enriched term negative regulation of nitrogen compound metabolic process (BP) (Appendix S6). Similarly, the term helicase activity (MF) was enriched in the DAPs that were higher at day 8 than 6 (Appendix S7).

There was an increase in abundance of protein transport proteins at pH 7.1 on day 10. Larvae at day 10 (compared to 8) had higher abundances of proteins enriched for the GO terms vesicle coat (CC) and transport vesicle (CC) (Appendix S7). The proteins associated with a possible upregulation of metabolism at pH 7.5 were absent in the analysis at pH 7.1

On day 12 postfertilization, there was a trend toward increases in abundance of proteins involved in metabolic processes in larvae at pH 7.1. Several proteins associated with biosynthetic processes contribute to the enriched term single‐organism biosynthetic process (BP) in cluster 5 (Appendix S6). In cluster 7, proteins that increased in abundance on day 12 contributed to the enriched terms phospholipid metabolic process (BP) and integral component of membrane (CC) (Appendix S6).

On day 17, cluster 43 proteins were enriched for the term sodium ion transmembrane transporter activity (MF) based on the inclusion of sodium‐ and chloride‐dependent glycine transporter and a sodium/hydrogen exchanger, the latter of which is involved in pH regulation (Appendix S6).

## 4 DISCUSSION

A global, high‐resolution proteomics survey was used to detect the molecular hallmarks of specific developmental transition points throughout the geoduck larval period. Across environmental rearing conditions, the abundances of basic molecular housekeeping proteins (e.g., proteins related to translation, ATP synthase, etc.) were maintained at steady levels. Geoduck larvae reared at different pH had different timing for developmental transitions. At pH 7.5, more proteins changed in abundance over time (types B‐E), suggesting more active regulation of physiological processes throughout development (Table 1). There were a higher number of proteins that did not change in abundance over days 6–17 postfertilization (type A) at pH 7.1 than at pH 7.5, which indicates less energetic demand to shift physiological resources to different processes at low pH.

Across both pH treatments, there were several protein categories that maintained stable abundances throughout the developmental time frame captured and that can be considered essential, housekeeping proteins. These categories included ribosomes, myosin and actin, proton‐transporting ATP synthase, protein folding, translation, and the citric acid cycle. In the clustered protein plots, these stably abundant protein clusters shared a higher percentage of proteins across pH treatments than other groups with time‐specific abundance patterns, indicating the pH levels used in this study did not have a significant impact on fundamental metabolic processes.

The most dominant signal of proteomic differentiation across the experiment was observed across developmental time, not by environmental pH. This highlights the degree to which numerous physiological processes are regulated during larval development. When considering proteomes across pH treatments in the NMDS, there was a trend of changes in abundance of proteins associated with various metabolic processes over time. This pattern suggests the importance of shifting larval metabolic needs over development.

### 4.1 Development at pH 7.5

During early geoduck larval development (6–8 days postfertilization), many molecular resources are dedicated to maintaining cellular integrity and growth. Thus, cluster and DAP analyses revealed higher abundances of cytoskeletal and membrane proteins at day 6 postfertilization compared to day 8. By day 8, there was an increase in abundance of proteins involved in cilia biogenesis, transport across cell membranes, and post‐translational modifications. The cilia biogenesis proteins are likely related to velum development. In other invertebrates, D‐veliger larval proteomes had relatively higher abundances of proteins involved in cell division, shape, and cellular differentiation than trochophores, suggestive of development of more complex morphology and velum development (Di et al., 2017; Huan, Wang, Dong, & Liu, 2012). Similarly, the abundance of cytoskeleton construction proteins increased from middle veliger through juvenile stages in the gastropod *Babylonia areolata* (Shen et al., 2018). These proteomic trends indicate continued cellular growth during this high‐growth early larval period, as well as protein turnover and cellular signaling and trafficking.

In the middle stages of larval development, there was continued evidence of cellular growth and evidence of increased processing of external resources associated with energetic demands of metamorphosis. The cytoskeletal protein signal at day 10 was dominated by tubulins, indicating that specific cytoskeletal proteins dominate at discrete developmental time points. Day 10 is also when there was a pronounced signal of glucose and fatty acid metabolism and protein degradation attributable to increased feeding. Proteins in cluster 10 (enriched for glucan metabolism) begin a steady increase in abundance starting at day 10, further indicating a relationship with metamorphosis and energetic demands. Other studies have demonstrated ingestion rate increases throughout larval development in the geoduck *Panopea globosa* (Ferreira‐Arrieta, García‐Esquivel, González‐Gómez, & Valenzuela‐Espinoza, 2015), which corresponds to the proteomic signatures observed. Changes in abundances of digestion enzymes were also observed in *B. areolata* as feeding strategies shifted throughout development (Shen et al., 2018).

Later developmental time points (days 14 and 17) in the geoduck mark a move toward the transition to the pediveliger stage and into competency for settlement. At this time point, there was a clear indication of physiological activity associated with translation, calcification, and protein turnover. On day 14 postfertilization, ribosomal proteins were elevated, indicative of increased translation. An increase in abundance of translation‐related proteins was detected in the proteomes of competent larvae compared to postsettlement juveniles of the Pacific oyster, *Crassostrea gigas* (Huan, Wang, & Liu, 2015). During this period, there was also an increased abundance of several proteins associated with shell deposition in geoduck. In the apple snail (*Pomacea canaliculata*) proteome, an increase in calcification‐related proteins concurrent with greater rates of calcification was noted in the transition to becoming juvenile snails (Sun, Zhang, Thiyagarajan, Qian, & Qui, 2010). By day 17, the ribosomal protein abundances had decreased, with an increase in proteins in the categories post‐translational modifications and transport. Taken together it would appear that we are observing the hallmarks of the transition of the larvae to a juvenile clam. There is surge in translation leading up to the production of proteins for shell formation and tissue restructuring. At this point, the larva would settle out of the water column and begin its benthic life stage. In fact, there is evidence of the neuronal signaling for this transition as high levels of proteins involved in neurotransmitter release were detected on day 17. Changes in expression of neurotransmitter pathway proteins and transcripts have also been noted in other invertebrate larvae as they approach settlement (Niu et al., 2016; Shen et al., 2018).

### 4.2 Development at pH 7.1

Larvae reared at low pH (7.1) were significantly smaller than geoduck larvae reared at pH 7.5 at day 17 postfertilization. This size discrepancy was observed as early as day 10 and the divergence increased throughout development (Figure 2), with a relative flattening of the growth curve in low pH larvae compared to control counterparts. Invertebrate larvae reared at low pH have typically been observed to be smaller than those at higher pH (e.g., Andersen, Grefsrud, & Harboe, 2013; Harney et al., 2016; Huo et al., 2019; Kapsenberg et al., 2018; Kurihara, Takamasa, Kato, & Ishimatsu, 2008; Padilla‐Gamiño, Kelly, Evans, & Hofmann, 2013; Parker, Ross, Raftos, Thompson, & O'Connor, 2011). Kurihara et al. (2008) targeted the beginning of pH‐influenced developmental delay at the gastrula stage, which is when the shell field is first formed. In *C. gigas*, this difference in size has been observed to be greater in the prodissoconch II (PD II) phase (Timmins‐Schiffman et al., 2013), as observed in the current study. In the mussel *Mytilus galloprovincialis*, larvae reared at low pH lacked the distinctive boundary between PD I and PD II shell material (Kurihara et al., 2008). It is possible that the developmental transition from PD I to PD II (a transition from amorphous calcium carbonate to an aragonitic shell; Medavkovic, Popovic, Grzeta, Plaxonic, & Hrs‐Brenko, 1997; Seung, Seong, & Cheong, 2006) at low pH is too energetically demanding to be executed successfully when the environment is not conducive to CaCO_3_ precipitation. Without information on developmental stage at the different time points, it is difficult to assess whether this slowed growth represents a delay in developmental rate or simply reduced size at low pH, both of which have been observed in invertebrate larvae. Given that larvae at pH 7.5 were larger and competent to settle before larvae reared at pH 7.1, the developmental delay hypothesis could be supported. In fact, metamorphosis in geoduck is known to be delayed by stress (Goodwin & Pease, 1989) and low pH (Huo et al., 2019). Developmental delay due to ocean acidification has been observed in other bivalve larvae, such as *C. gigas* and *M. galloprovincialis* (De Wit, Durland, Ventura, & Langdon, 2018; Frieder et al., 2016; Kurihara et al., 2008; Timmins‐Schiffman et al., 2013). It is also possible that the larger size and faster development at pH 7.5 were due in part to greater mortality rate at the higher pH, possibly releasing constraints of density effects on larval size. The larvae were reared at standard densities according to hatchery procedures, thus support of the density stress hypothesis is unlikely.

The other marked difference in response between the two pH treatments was a disconnect in the timing of specific molecular events during larval development. This disconnect was also observed in *C. gigas* larvae reared at different pH and attributed to a potential delay in development (i.e., sampling of different developmental time points) (Harney et al., 2016). Whereas many housekeeping proteins maintained stable abundances across both pH treatments, the number and function of proteins that changed in abundance at specific developmental time points differed (Table 1). When comparing proteins with shared membership between clusters at different pH, none of the nonhousekeeping clusters shared >50% of their protein members, indicating discrete patterns of protein abundance based on environmental pH. For example, at pH 7.5, there was a large signal of ribosomal protein upregulation around days 12 and 14, but a similar signal occurred around days 6 and 8 at pH 7.1. Due to the frequency of sampling for proteomics, it is likely that we missed some transitions in timing of developmental events; however, the proteomic profiles between the two pH were different enough to suggest that we captured large‐scale impacts of pH on developmental timing. These contrasts suggest distinct developmental paths based upon environmental conditions, with at least one phenotypic outcome being significantly smaller larval size.

Early developmental time points for geoduck reared at pH 7.1 were dominated by higher abundances of proteins involved in DNA replication, transcription, translation, and signaling, contrasted with at pH 7.5 higher abundances of cytoskeletal and membrane proteins. At day 6, there was a strong proteomic signal of ribosomal proteins, proteins that support transcription, and cellular signaling. Unhindered protein synthesis under low pH conditions has been observed in *C. gigas* (Frieder et al., 2016) and maintenance of translation‐related proteins may be further evidence that ocean acidification does not impact this essential process. At day 8, the molecular developmental signals were still diverged between the two pH treatments, with an increase in abundance of DNA replication and translation proteins at pH 7.1.

Days 10 and 12 of development at pH 7.1 revealed proteomic evidence of cytoskeletal modification, metabolism, and cellular growth. There were similar changes in these general proteomic categories at these time points at pH 7.5; however, the specific proteins involved differed between the pH treatments. On day 10 at both pH, proteins involved in the cytoskeleton were at increased abundance related to shifts in cellular morphology and growth at this time point. Abundances in cytoskeletal proteins have been observed to change between larval stages in other invertebrates (Di et al., 2017; Huan et al., 2015; Shen et al., 2018). Increased *p*CO_2_ has a documented effect on the level of cytoskeletal gene transcripts and proteins. Gene transcripts for cytoskeletal proteins decreased at elevated *p*CO_2_ in urchin pluteus larvae (Padilla‐Gamiño et al., 2013) and protein abundances of structural molecules decreased in abundance in *Crassostrea hongkonensis* pediveligers (Dineshram et al., 2015) and in *C. gigas* D‐hinge larvae (Harney et al., 2016). Proteins involved in increased protein production were at increased abundance at pH 7.1, with protein transport and translation proteins at increased abundances, compared to protein degradation and post‐translational modification proteins at increased abundances at pH 7.5. There were also signals of an upregulation of metabolism at both pH; however, this signal was more pronounced at days 10 and 12 at pH 7.5 and at day 12 only at pH 7.1 and involved very different metabolic pathways at both pH. At pH 7.1, there were increases in abundances of proteins involved in biosynthesis and phospholipid metabolism. The differences in metabolism‐related proteins between the two pH suggest different pathways of resource breakdown and digestion. Low environmental pH has been found to negatively affect sea urchin larvae digestion by decreasing the alkalinity of their stomachs (Stumpp et al., 2013), which could also impact geoduck and thus change the molecular signals of metabolism.

In later stages of larval development, there was strong signal of calcification‐related proteins on day 14 postfertilization at pH 7.1, with increased abundances of several mucin isoforms, IgGFc‐binding protein, and chitotriosidase, similar to the signal observed in pH 7.5 larvae. This correlates with calcification in the prodissoconch II stage of development, which is characterized by a change in structure of shell crystals (Medavkovic et al., 1997; Seung et al., 2006) necessarily supported by shifts in the proteome. Given that larvae from pH 7.5 settled sooner than those at 7.1 at least some of the proteomic signals that were important at pH 7.5 underlie the physiological shifts necessary for metamorphosis and competency. Some of the protein categories not detected in our analysis of pH 7.1 were neurotransmitter release, membrane budding, and carbohydrate metabolism. It is possible that the low pH larvae were not in the appropriate metabolic state to transmit the intercellular signals to begin metamorphosis.

By following invertebrate larval development over two weeks, this dataset reveals that larvae reared at low pH experience disruptions to their molecular physiology in terms of timing of proteomic physiological changes during development. These disrupted patterns observed in the proteome are likely a result of the energetic toll placed on the larvae as they grow and calcify at a low pH that can negatively impact multiple physiological processes (e.g., Stumpp et al., 2013; Waldbusser et al., 2013). Essential, housekeeping proteins were maintained at stable levels providing insight into the possible prioritization of energetic resources under stressful environmental conditions. Despite the somewhat coarse temporal resolution of our sampling design, clear differences between the two pH treatments were still apparent. In geoduck veligers, low pH alters molecular pathways underlying important physiological processes, such as cellular structure and integrity, metabolism, protein turnover, and calcification. Ultimately, the low pH exposed geoduck larvae were smaller, abnormal in morphology, developmentally delayed, and did not settle by day 19 postfertilization as observed at pH 7.5. Similar results were observed in the geoduck *P. japonica* in water acidified by hydrochloric acid (Huo et al., 2019). Even though geoduck clams live in a naturally lower pH environment, their larval stages are not resilient to the impacts of ocean acidification. This may be in part because local pH in northeastern Pacific habitats increases during the summer larval period (Lowe, Bos, & Ruesink, 2019) so there has been no evolutionary impetus to adapt to low pH during the pelagic larval stage. Going forward, geoduck populations may be at risk of lower larval survival and recruitment, but we also must consider adaptation, species interactions, and environmental memory to better understand ecosystem impacts.

## CONFLICT OF INTEREST

The authors have no conflicts of interest to report.

## AUTHOR CONTRIBUTIONS

E. T. S. was involved in data collection and was responsible for data analysis and interpretation and writing the manuscript. J. G. was responsible for sample prep for mass spectrometry, mass spectrometry data collection, and assisted with data interpretation and drafting of the manuscript. R. E. T. was responsible for carrying out the experiment in the shellfish hatchery and assisted in manuscript preparation. B. V. was responsible for experimental design and assisted in drafting of the manuscript. B. E. supervised the experiment at the hatchery and was involved in drafting of the manuscript. S. B. R. was responsible for experimental design with B. V. and contributed to data interpretation and drafting of the manuscript. All authors approved the submitted manuscript.

### Open Data Badges


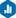


This article has earned an https://openscience.com for making publicly available the digitally‐shareable data necessary to reproduce the reported results. The data is available at https://www.ebi.ac.uk/pride/archive/projects/PXD013667.

## Data Availability

All proteomics data are available on the ProteomeXchange Consortium via the PRIDE partner repository with the dataset identifier PXD013667.
