# Supplementary figures and images for: Dynamic response in the larval geoduck (Panopea generosa) proteome to elevated pCO2
Source: Ecol Evol. 2019 Dec 6;10(1):185–97. doi: 10.1002/ece3.5885 (PMC6972802; doi:10.1002/ece3.5885)

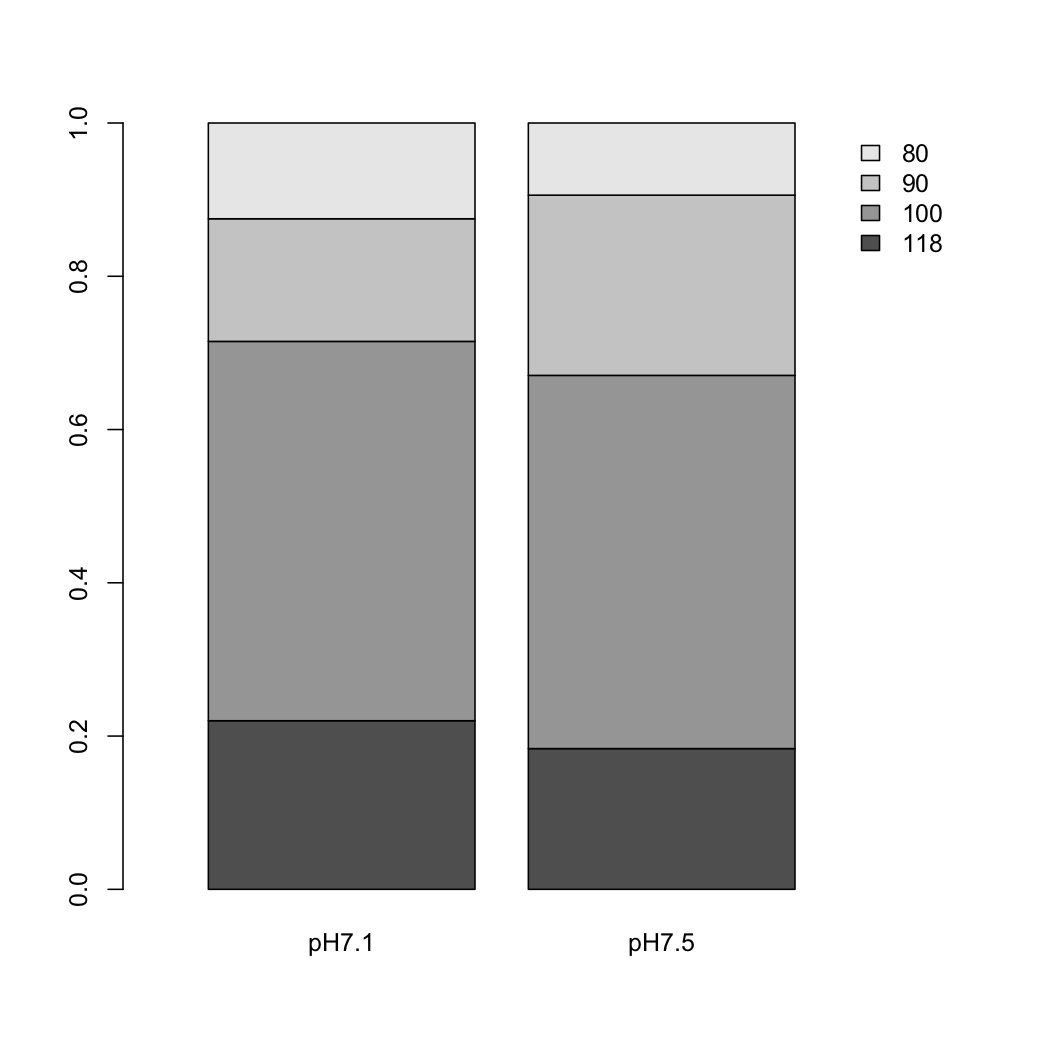

Supplement: Supplementary file 1 [file ECE3-10-185-s001.jpg]
