# Supplementary material for: Dynamic response in the larval geoduck (Panopea generosa) proteome to elevated pCO2
Source: Ecol Evol. 2019 Dec 6;10(1):185–97. doi: 10.1002/ece3.5885 (PMC6972802; doi:10.1002/ece3.5885)
Supplement: Supplementary file 9 [file ECE3-10-185-s009.docx]

Appendix 1: Proportion of larvae in each size class (µm) on day 10 at pH 7.1 and 7.5.

Appendix 2: Eigenvector loadings for the nonmetric multidimensional scaling analysis. Each protein (eigenvector) is listed with its eigenvector loadings on axis MDS1 and MDS1, along with an r^2^ and p-value.

Supporting Information 1: Larval mass conversion used by Taylor Shellfish Hatchery. For each screen size, the number of grams equal to 1 million larvae is provided.

Supporting Information 2: Proteomics results summary data. The first column contains the identifiers of 6328 proteins identified in the dataset. Subsequence columns with headers that begin with “G” (e.g., “G7_2”) contain normalized spectral abundance values (a proxy for protein abundance) for each mass spectrometry run in the project. These are followed by 2 columns that contain the sum of NSAF across all samples from pH 7.1 and pH 7.5. Next are 2 columns with the cluster number that each protein clustered into for the hierarchical cluster analysis. The “uniprotID” column contains the UniProt annotation for each protein. The last set of columns contains an asterisk if a given protein was differentially abundant in the QSpec analysis.

Supporting Information 3: Enrichment analysis results for protein clusters for pH 7.5 larvae (Figure 4) for Biological Process (BP), Cellular Component (CC), and Molecular Function (MF). Each tab in the workbook contains the enrichment results for a given cluster. Results include the GO term Accession Number, the GO term name, a p-value associated with the GO term’s enrichment in the dataset, and the proteins contributing to the enrichment of the GO term.

Supporting Information 4: Enrichment analysis results for protein clusters for pH 7.1 larvae (Figure 5) for Biological Process (BP), Cellular Component (CC), and Molecular Function (MF). Each tab in the workbook contains the enrichment results for a given cluster. Results include the GO term Accession Number, the GO term name, a p-value associated with the GO term’s enrichment in the dataset, and the proteins contributing to the enrichment of the GO term.

Supporting Information 5: Enrichment analysis results for differential abundance analysis for pH 7.1 larvae for Biological Process (BP), Cellular Component (CC), and Molecular Function (MF). Each tab in the workbook contains the enrichment results for a given group of differentially abundant proteins (DAPS). Results include the GO term Accession Number, the GO term name, a p-value associated with the GO term’s enrichment in the dataset, and the proteins contributing to the enrichment of the GO term.

Supporting Information 6: Enrichment analysis results for differential abundance analysis for pH 7.5 larvae for Biological Process (BP), Cellular Component (CC), and Molecular Function (MF). Each tab in the workbook contains the enrichment results for a given group of differentially abundant proteins (DAPS). Results include the GO term Accession Number, the GO term name, a p-value associated with the GO term’s enrichment in the dataset, and the proteins contributing to the enrichment of the GO term.
